# Individual Differences in Children’s Development of Scientific Reasoning Through Inquiry-Based Instruction: Who Needs Additional Guidance?

**DOI:** 10.3389/fpsyg.2020.00904

**Published:** 2020-05-14

**Authors:** Erika Schlatter, Inge Molenaar, Ard W. Lazonder

**Affiliations:** Behavioural Science Institute, Radboud University, Nijmegen, Netherlands

**Keywords:** scientific reasoning, inquiry-based learning, elementary education, individual differences, instructional guidance

## Abstract

Scientific reasoning involves a person’s ability to think and act in ways that help advance their understanding of the natural world. Young children are naturally inclined to engage in scientific reasoning and display an emerging competence in the component skills of, for example, hypothesizing, experimenting and evaluating evidence. Developmental psychology research has shown that same-age children often differ considerably in their proficiency to perform these skills. Part of this variation comes from individual differences in cognition; another part is due to the fact that the component skills of scientific reasoning emerge at a different age and mature at a different pace. Significantly less attention has been paid to children’s capacity to improve in scientific reasoning through instruction and deliberate practice. Although elementary science lessons are generally effective to raise the skill level of a group of learners, not all children benefit equally from the instructional treatment they receive. Knowing what causes this differential effectiveness is important as it can inform the design of adaptive instruction and support. The present study therefore aimed to identify and explain how fifth-graders (*N* = 138) improve their scientific reasoning skills over the course of a 5-week inquiry-based physics unit. In line with our expectations, significant progress was observed in children’s achievements on a written scientific reasoning test, which was administered prior to and after the lessons, as well as in their responses to the questions and assignments that appeared on the worksheets they filled out during each lesson. Children’s reading comprehension and mathematical skillfulness explained a portion of the variance in children’s pretest-posttest gain. As these overall results did not apply equally to all component skills of scientific reasoning, we recommend science teachers to adapt their lessons based on children’s past performance in reading and math *and* their actual performance of each scientific reasoning skill. The orchestration and relative effectiveness of both adaptive science teaching approaches is an interesting topic for future research.

## Introduction

Elementary science education acquaints children with fundamental science concepts such as buoyancy, motion and electricity, and introduces them to the basics of doing scientific research. School science lessons make ample use of inquiry-based teaching methods, which enable children to learn to think and act in ways that help advance their understanding of the natural world (Kind and Osborne, 2017). This ability is commonly referred to as *scientific reasoning* and involves the skills of hypothesizing, experimenting and evaluating evidence (Klahr and Dunbar, 1988; Zimmerman, 2007; Kind, 2013). The main purpose of this study was to investigate how these skills develop during an inquiry-based science unit, and which cognitive characteristics predict children’s level of skillfulness at the end of the unit.

The teaching and learning of scientific reasoning is a challenging task for both teachers and children. One complicating factor is that considerable individual variation exists among children in the same classroom (Koerber et al., 2015; Lazonder et al., submitted). To complicate matters further, the component skills of scientific reasoning are known to emerge at different ages and develop at a different pace (Piekny and Maehler, 2013). Kindergartners already show some initial proficiency in basic experimentation and evidence evaluation skills whereas the more difficult skill of hypothesizing usually starts developing around the age of 12. These accumulating differences point to a clear need for adaptive instruction, but until now few evidence-based guidelines for designing and delivering adaptive and age-appropriate science lessons seem to exist.

In working toward establishing such guidelines, the present study sought to unveil whether and to what extent the progress monitoring data available in schools can help predict differences in children’s ability to learn scientific reasoning. Many schools have access to rich data records that portray children’s developmental trajectories in the foundation skills of language and math. As these skills are related to children’s scientific reasoning *performance* (e.g., Tajudin and Chinnappan, 2015; Van de Sande et al., 2019), it seems worthwhile to investigate their predictive powers for the *development* of scientific reasoning in an instructional setting. Additionally, process data collected during the lessons was analyzed in order to identify key learning moments. The insights that result from these investigations can help teachers to respond adequately to individual differences during their science lessons.

## Theoretical Framework

### Development of Scientific Reasoning

Scientific reasoning is a multidimensional process that consists of several component skills. Although scholars diverge on the definition and labeling of these skills (for an overview, see Pedaste et al., 2015), consensus seems to exist on the core skills of hypothesizing, experimenting and evaluating evidence (Zimmerman, 2007; Kind, 2013). Even though these are difficult skills even for adults (Zimmerman, 2007), children at the pre-school age already show some emerging proficiency in an skills (Sodian et al., 1991; Piekny et al., 2014; Van der Graaf et al., 2015; Köksal-Tuncer and Sodian, 2018; Van der Graaf et al., 2015) that develops steadily but slowly during the elementary school years (Kuhn, 2002; Piekny and Maehler, 2013; Koerber et al., 2015).

Although developmental growth occurs in all component skills, their emergence and pace of development varies. Experimenting is relatively easy to learn and even young children can be rather proficient in the basics of experimentation (Cook et al., 2011; Van der Graaf et al., 2018; Schalk et al., 2019; Van der Graaf et al., 2018). Hypothesizing is more difficult for children to learn (Piekny and Maehler, 2013; Schlatter et al., 2019) and this skill generally emerges late and develops slowly. Results regarding evidence evaluation are mixed. First-graders can already draw correct conclusions from perfectly covarying data (Koerber et al., 2005; Piekny and Maehler, 2013; Van der Graaf et al., 2018), but the evaluation of non-perfect covarying evidence in light of hypotheses remains difficult throughout elementary school (Piekny and Maehler, 2013).

In addition to this variation across component skills, same-age children are not equally well versed in scientific reasoning either. In a large-scale cross-sectional study using written tests in grades 2 to 4, Koerber et al. (2015) distinguished between naïve, intermediate, and advanced conceptions of scientific reasoning. Although older children generally had a more sophisticated view, all three proficiency levels were present in all participating grade levels. The cross-sectional results of Piekny and Maehler (2013) further suggest that these inter-individual differences increase with age in all component skills. For example, both the means and standard deviations of hypothesizing were low in Kindergarten, but increased from grade 1 onward. These findings indicate that, although children’s hypothesizing skills undergo a steady growth, the variation among peers grows accordingly. Thus, children improve in scientific reasoning with age, but not all children improve at the same pace. Acknowledging these individual differences alongside the dissimilar difficulty levels of the component skills is vital for good science education.

### Predictors of Scientific Reasoning

Several studies have examined what accounts for observed differences in children’s scientific reasoning (Siler et al., 2010; Mayer et al., 2014; Wagensveld et al., 2014; Koerber et al., 2015; Wagensveld et al., 2014). Reading comprehension was a significant predictor in all these studies, whereas cognitive characteristics such as spatial reasoning, problem solving skill, and general intelligence had a less prominent and less consistent impact. Although mathematical skillfulness has been shown to correlate with scientific reasoning (Bullock and Ziegler, 1999; Koerber and Osterhaus, 2019), to the best of our knowledge, no studies have examined whether mathematical skillfulness predicts scientific reasoning. This seems remarkable because scientific reasoning tasks often require children to handle numerical data (Kanari and Millar, 2004), which, in turn, could be the reason why national curriculum agencies consider mathematical skillfulness as a prerequisite for scientific reasoning instruction (e.g., van Graft et al., 2018; Wong, 2019).

Research into the predictors of scientific reasoning either treated scientific reasoning as a unitary construct or focused on one of its components, with experimenting being the most widely studied skill. Studies that assess and report children’s performance on multiple component skills are clearly underrepresented in the literature and, as a consequence, little is known about how well reading comprehension and mathematical skillfulness predict children’s proficiency in separate scientific reasoning skills. Initial evidence suggests that both predictors may have differential effects. Schlatter et al. (2019) established that reading comprehension predicts performance on all subskills except hypothesizing, whereas Van de Sande et al. (2019), who administered a written test, found a strong explanatory effect of reading comprehension on this skill and lower impacts on experimenting and drawing conclusions. Osterhaus et al. (2017) found no effect of language abilities on experimenting–although it did influence children’s understanding of the nature of science. These findings, although apparently contradictory, emphasize the importance of analyses at the subskill level. However, as these studies examined children’s scientific reasoning *performance*, research still has to determine whether and to what extent reading comprehension and mathematical skillfulness affect and predict children’s *learning* of the component skills of scientific reasoning in regular science classrooms.

### In-School Learning of Scientific Reasoning

Studies examining the development of scientific reasoning in an instructional setting predominantly target children’s ability to design and conduct controlled experiments. The natural development of this skill can be boosted in a short period of time through various instructional methods that often yield long-term effects. Implicit methods such as giving hints to focus the investigation on a single variable (Kuhn and Dean, 2005), dividing the research question in single-variable subquestions (Lazonder and Wiskerke-Drost, 2015), providing scaffolds (van Riesen et al., 2018) or opportunities for sustained practice (Schalk et al., 2019) all improve children’s experimentation skills. Explicit instructional methods that explain and/or demonstrate the design of controlled experiments have similar benefits (Chen and Klahr, 1999; Lorch et al., 2017). A recent meta-analysis substantiated that implicit and explicit methods are equally effective for promoting experimenting skills (Schwichow et al., 2016).

The skills of hypothesizing and evaluating evidence have less often been trained in isolation, but are included in integrated studies of scientific reasoning, often using microgenetic designs. In a 3-year longitudinal study, Kuhn and Pease (2008) found that repeated practice alone promotes children’s evidence evaluation skills throughout grades 4 to 6. Hypothesizing skills improved only when children were in sixth grade–despite frequent opportunities for practice in the preceding years–and individual change patterns in both skills varied considerably, with relapses to old, less-effective routines. More explicit instructional support can accelerate children’s natural pace of development. Greven and Letschert (2006) showed that sixth-graders who merely investigated a multivariable system did improve their ability to evaluate evidence over the course of a 5-week inquiry-based lesson series. However, significantly higher learning gains were observed in children who received additional prediction practice exercises (that focused their attention on integrating the impact of multiple variables) or explicit instruction on the concept of multivariable causality.

To conclude, the cited studies exemplify that even short instructional interventions can promote children’s scientific reasoning. Prolonged opportunities for practice have similar beneficial effects but seem more difficult to realize in regular science classrooms. Striking the right balance between independent practice and instructional guidance thus seems a major challenge elementary science teachers have to meet. This orchestration of instructional support is complicated further by the substantial variation across the component skills and among same-aged children. As a large share of this variance remains unexplained, the present study aimed to describe and explain children’s development of scientific reasoning skills in inquiry-based classrooms.

### Research Questions and Hypotheses

Previous research has shown that the component skills of scientific reasoning are not equally well developed and learned in upper-elementary science classes. Although these individual differences are explained in part by children’s cognitive characteristics, with reading comprehension being the most robust predictor, questions remain as to how the core scientific reasoning skills of hypothesizing, experimenting and evaluating evidence develop in an instructional setting, and how developmental differences can be adequately accommodated by elementary science teachers. The present study therefore aimed to find out:

(1) To what extent fifth-graders improve their scientific reasoning skills during a 5-week inquiry-based unit;
(2) Whether observed differences in learning gains are contingent on children’s reading comprehension and mathematical skillfulness; and
(3) Whether there are any key moments during this lesson series where children make marked progress in their application of the component scientific reasoning skills.

These research questions were examined in a sample of Dutch fifth-graders, who engaged in 5 weekly science lessons. Each lesson revolved around a hands-on investigation that enabled children to practice the component skills of hypothesizing, experimenting and evaluating evidence. Children’ investigations were guided by worksheets and a whole-class introduction to the steps of the inquiry cycle. Learning gains were assessed by a written scientific reasoning pre-test and post-test. Learning process data were collected from the children’s worksheets, whereas children’s scores on standardized progress monitoring tests of reading comprehension and mathematics were obtained from the schools’ administration.

Hypotheses regarding the first research question predicted that children would make progress in all scientific reasoning skills–but not to the same degree. As previous research has shown that hypothesizing and evaluating evidence is largely beyond fifth-graders’ reach, these skills were expected to improve marginally and comparably in just five lessons. Experimenting on the other hand is known to be relatively easy so children might already be rather adept in this skill and, hence, have less opportunity for improvement compared to the other skills. However, in absence of a national science curriculum and instigated by recent policy measures, many Dutch elementary schools are just beginning to systematically incorporate science in their curriculum (Inspectie van het Onderwijs, 2017), a rival hypothesis therefore predicted that children’s experimentation skills are initially lower than expected based on international benchmarking studies, but will improve more rapidly over the course of the five lessons than the other component skills – a result more often observed in intervention studies (Lorch et al., 2014; Peteranderl, 2019).

The second set of hypotheses related to the prediction of learning progress. Even when no overall learning gain is found, part of the sample could have made significant progress. To explain such possibly differential progress, two predictor variables were used: reading comprehension and mathematical skillfulness. Previous studies have shown that the former consistently predicts individual differences in scientific reasoning performance. We therefore felt it safe to assume that reading comprehension would explain learning gains in all three component skills. Evidence regarding the impact of children’s mathematical skillfulness is limited, but existing studies suggest that ‘being good with numbers’ serves as an advantage when interpreting the numerical outcomes of science experiments (Bullock and Ziegler, 1999; Koerber and Osterhaus, 2019). Children’s mathematical skillfulness was therefore expected to predict learning gains in evidence evaluation.

Thirdly, children’s worksheets were scrutinized for evidence of possible growth spurts in children’s learning of the three component scientific reasoning skills. In absence of any theoretical and empirical underpinnings, no explicit hypothesis was made regarding the outcome of this analysis.

## Materials and Methods

This study was carried out in accordance with the recommendations of the ethics code for research with human participants in the social and behavioral sciences, as agreed upon by the Deans of Social Sciences in the Netherlands. The protocol was approved by the Ethics Committee of the Faculty of Social Sciences at Radboud University, under number 2018-074R1. Descriptive data (gender and year of birth) were collected anonymously while other data (pre- and post-test, worksheets and standardized test scores) were pseudonymized.

### Participants

In the Fall of 2018, eight fifth-grade classes (in Dutch: ‘groep 7’) from six schools in the central and northern part of the Netherlands participated in this study. All children in these classrooms received five 1-hr lessons as part of their regular science curriculum. Passive parental consent was sought with the exception of one school, whose principal preferred active parental permission for participation. Children with parental consent (*N* = 154) also took a scientific reasoning pre- and post-test; the worksheets they filled out during the lessons were collected for analysis, and their progress monitoring scores on standardized tests of reading comprehension and mathematics were obtained from the school. Sixteen children were excluded from the analyses, either because they missed more than one lesson, had not taken the pre- or post-test, or because their reading and math progress monitoring records could somehow not be obtained. The final sample thus consisted of 138 participants (55% boys) who were between 8 and 12 years of age; the majority of the sample was 10 years old.

### Materials

#### Lesson Materials

**FIGURE 1:**
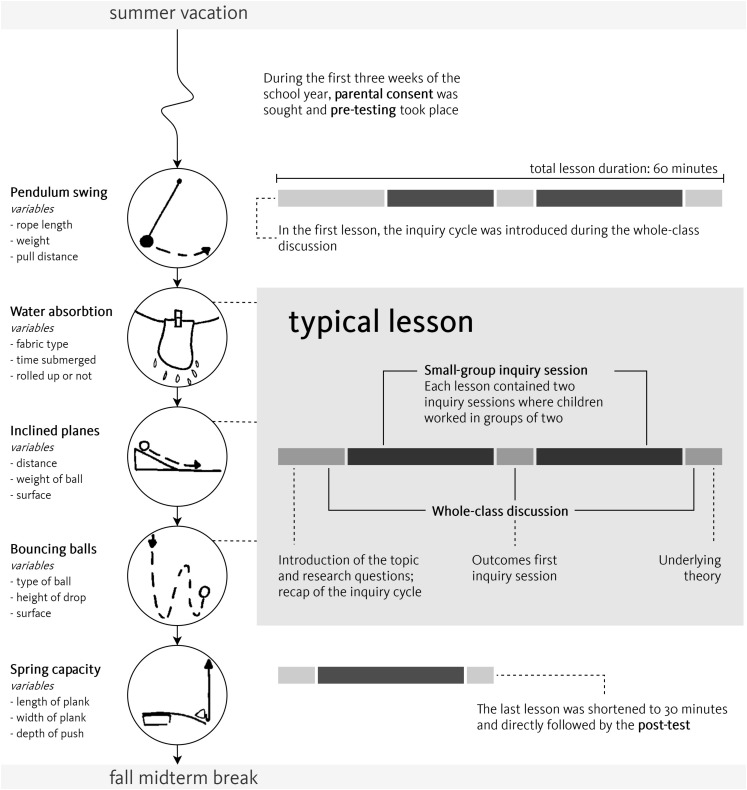
Outline of the lesson series.

Children engaged in five science lessons that addressed elementary-school physics topics (see Figure 1) through an inquiry-based teaching approach, taught by the first author. All lessons were structured similarly and contained two types of activities: whole-class discussion and small-group work. Each lesson started with a plenary introduction (lesson 1) or refresher (lessons 2–5) of the inquiry cycle, and introduced children to the topic of inquiry. Children then started their first inquiry, which they completed in 20 min. In order to mimic authentic classroom practice, children conducted their investigation in dyads, which they formed themselves on an ad-hoc basis. As children chose their learning partners based on friendship rather than academic achievement and partnerships rotated during the lesson series, the chances of any systematic bias due to group formation were assumed to be negligible. The first inquiry was wrapped up during a short whole-class discussion that addressed questions such as ‘who found an answer to the research question?’ and ‘who found a different result than hypothesized?’ After the second 20-min inquiry cycle, children reconvened for a final whole-class discussion of the outcomes of the inquiry and the underlying physics principles.

The lessons were designed to practice four scientific reasoning skills: hypothesizing, experimenting, interpreting data, and drawing conclusions. Each lesson centered around a different subject-specific topic (see Figure 1) that children could learn about through experimentation. All experiments had three dichotomous input variables and one continuous output variable. For example, the pendulum swing experiment enabled children to manipulate the length of the rope (long or short), the weight of the pendulum (heavy or light), and the amplitude (far or close). Children used a stopwatch to measure the time it took to make five swings. In a typical lesson, the experimental equipment would be used during two inquiry sessions that were structured according to the inquiry cycle and enabled children to investigate two distinct research questions.

All inquiry sessions were supported by worksheets (see Supplementary Appendix 1) that assisted children in performing the four scientific reasoning skills of the inquiry cycle. This guidance consisted of a pre-specified research question and several scaffolds, that structured children’s inquiry without explicitly instructing them what to do and why. Specifically, children could complete sentence starters to make their hypotheses and conclusions, and complete pre-structured tables to set up an experiment and interpret the results. The worksheets contained text and pictures that served to remind children of the research question and the variables under investigation (for an overview of inquiry topics and variables, see Figure 1).

This amount of support, defined by Bell et al. (2005) as *guided inquiry*, purposefully constrained the number of strategies children could apply, and has been shown to facilitate the learning of scientific reasoning skills (e.g., van Riesen et al., 2018). For example, providing a research question minimized the risk of children conceiving a research question that could not be investigated, while still providing them with a fair degree of autonomy in their inquiry. The worksheets thus had a dual purpose: in addition to being a supportive device, they served as a measure of children’s progress in scientific reasoning. Even though children conducted their investigations in dyads, they wrote down what they themselves thought to be the best hypothesis, experiment, interpretation of the data they gathered, and conclusion. As such, this process data could be used to identify where additional support was needed, and thus inform future research on adaptive science instruction.

Supplementary to the worksheets, more elaborate support was given during whole-class discussions and to individual children who indicated they were struggling with the assignments. Children who struggled were first prompted to write down what they thought was best. If they were still hesitant to work on the worksheet, guidance was slowly increased following the protocol in Supplementary Appendix 2. In practice, children rarely asked for help and no child asked help repeatedly for the same component skill. During the whole-class discussions, children were invited to share what they remembered about the inquiry cycle, what they found out during their investigations and what they thought were the underlying scientific principles. If answers were limited (e.g., ‘we found that it made a difference’), children were encouraged to provide more detail (e.g., ‘can you explain more precisely what you found?’).

#### Scientific Reasoning Inventory

Children’s scientific reasoning skills were assessed at pre- and post-test using the Scientific Reasoning Inventory (SRI; Van de Sande et al., 2019), a pencil-and-paper test consisting of 24 multiple-choice items with three to four answer options each. Items were thematically embedded in five cover stories that were meaningful and appealing to children, such as the living conditions of wildlife and sports activities.

During the original validation of the SRI, three scales emerged: hypothesis validation (which included data interpretation), experimentation and drawing conclusions (Van de Sande et al., 2019). Confirmatory factor analysis was performed and results, including the comparative fit index (CFI), root mean square error of approximation (RMSEA) and standardized root mean square residual (SRMR) are reported below. In our pre-test data, a single-factor solution had a rather poor fit, χ^2^(252) = 437.04, *p* < 0.001, CFI = 0.608, RMSEA = 0.067, SRMR = 0.081. The original three-factor model had a better fit, χ^2^(249) = 354.99, *p* < 0.001, CFI = 0.776, RMSEA = 0.051, SRMR = 0.075, and the four-factor model, with data interpretation as a separate factor, yielded comparable fit statistics, χ^2^(246) = 352.15, *p* < 0.001, CFI = 0.775, RMSEA = 0.051, SRMR = 0.074. While the improvement from the single-factor model to the three-factor model was significant, χ^2^_diff_(3) = 82.046, *p* < 0.001, the improvement from the three-factor model to the four-factor model was not, χ^2^_diff_(3) = 2.841, *p* = 0.417. We therefore decided to use the original three scales in the analyses. As a consequence, there was no one-on-one match between the SRI-scales and the skills addressed by the worksheets. Specifically, hypothesizing and interpreting outcomes were separate skills on the worksheets but combined in one SRI-scale, which we labeled ‘hypothesis-evidence coordination.’

This *hypothesis-evidence coordination* scale (9 items, α_pretest_ = 0.66, α_posttest_ = 0.74), consisted of two types of items. Five items presented children with four research questions, and asked them to select the question that best matched the research purpose described in the cover story. The nature of these items closely resembled the way in which the skill of hypothesizing was addressed during the lessons. Four additional items measured children’s ability to interpret a table with research data. These questions related to the skill of interpreting data as was addressed during the lessons. Although these nine items loaded on the same scale in the SRI, they were practiced separately during the intervention because they took place in a different stage of the inquiry cycle.

The second scale, *experimenting* (7 items, α_pretest_ = 0.47, α_posttest_ = 0.81), required children to select the best experiment based on the cover story. Each item presented children with three experimental designs with either two variables (2 items) or three variables (5 items). For each experiment only one experimental setup allowed for valid causal conclusions. The other experiments were either confounded, did not change any variables, or were controlled but did not manipulate the target variable.

Items on the third scale, *drawing conclusions* (8 items, α_pretest_ = 0.64, α_posttest_ = 0.77), contained two premises and a question about those premises children could answer with ‘yes,’ ‘no,’ or ‘maybe.’ These syllogisms were embedded in the overarching cover story. For example, one of the syllogisms in the sports storyline was: ‘All children who will go rowing, are wearing shorts. Anna will go rowing. Is she wearing shorts?’

#### Reading Comprehension and Mathematical Ability

Most schools in the Netherlands participate in the student monitoring program of the National Institute for Educational Testing and Assessment [Stichting Cito Instituut voor Toetsontwikkeling]. This program includes standardized assessments of children’s cognitive abilities, which are administered twice a year. The tests of reading comprehension and mathematical skillfulness were used in the present study.

The reading comprehension test provided children with different types of texts, such as short stories, newspaper articles, advertisements and instructional manuals (Weekers et al., 2011). The test consisted of 55 multiple-choice items that, for example, required children to fill in the blanks, explain what a particular line in the text meant or choose an appropriate continuation of a story. The mathematics test had children solve 96 multiple-choice and open-ended problems that were presented either with or without context (Hop et al., 2017). Contextualized problems consisted of a short text in which the problem was outlined and a supporting picture. In problems without context, children would only be presented with the numerical operations. Sample items of both tests can be found in Supplementary Appendix 3.

The monitoring program provides raw scores as well as a proficiency score (I-V, with I being the highest level and V the lowest). The latter can be used to meaningfully compare scores across different versions of the monitoring program. Because all participating schools used the same student monitoring program, but not all schools used the same version, these proficiency scores were used as predictor variables. As such, the association between children’s scientific reasoning and their proficiency in reading comprehension and mathematics could be assessed without burdening children with more tests. In order to improve interpretability, proficiency scores were recoded so that 1 represented the lowest proficiency and 5 the highest proficiency.

#### Worksheet Scoring

The worksheets served a dual purpose in this study. In addition to being a supportive device, they were used as a process measure of children’s learning. To this end, the worksheets of all five lessons were made as comparable as possible, differing only with regard to subject content (i.e., names of variables and images directly related to the subject-specific content). The questions and scaffolds were identical throughout the lesson series.

Worksheets were coded for each component skill (i.e., hypothesizing, experimenting, interpreting data, drawing conclusions). For each skill a maximum of 3 points was awarded, resulting in a maximum of 12 points per worksheet (see Table 1 for the coding scheme). *Hypotheses* were classified according to their level of specificity using the criteria proposed by Lazonder et al. (2010). Given the young age group in the current study, the definition of a fully-specified hypothesis was slightly altered: it included the variables involved and a prediction of the direction of effect. *Experimenting* was assessed from children’s use of the control-of-variables strategy (CVS; Chen and Klahr, 1999). It is important to note that this was not an all-or-nothing evaluation: even if the CVS was not applied, some points could still be awarded depending on the severity of the misconceptions (Peteranderl, 2019). At the very least, children had to understand the need for contrast, so a confounded experiment still received one point, whereas an experiment in which no variables were changed received zero points. The worksheet assignment for *interpreting data* consisted of two parts. The first part was a yes/no question that asked children whether they had observed a difference in outcomes between the two values of the focal variable. If the inference matched their data, one point was awarded. This inference should ideally be made based on multiple iterations of the same experiment. However, data gathered by children can be complex and messy (Kanari and Millar, 2004) and if this was the case, the single comparison was evaluated as a check. In the second part, children were asked to justify their inference. Two more points were awarded if children stated that they used the data to make this inference (a verbal statement of (non)covariation; Moritz in Ben-Zvi and Garfield, 2004) and/or explained what caused the result they found. *Conclusions* were, like hypotheses, evaluated in terms of their specificity (Lazonder et al., 2010). In addition to the criteria described above, the effect children mentioned in their conclusion had to match the data they gathered.

**TABLE 1:** Coding scheme.

Skill	Evaluation criteria	Example
Hypothesizing	- An **effect** was described - The **direction** of the effect was described - The **variables involved** were described	‘I think it makes a difference’ (1 point: effect described) ‘I think the surface matters for the number of bounces (2 points: effect and variables described; no direction) ‘I think there will be more bounces on a hard surface’ (3 points)
Experimenting	- **Comparison** is possible: at least one variable has been changed - **Fair comparison** is possible: only one variable has been changed - Experiment **aligns with the research question:** focal variable has been changed	Confounded experiment (1 point: comparison possible) Controlled experiment on non-focal variable (2 points) Controlled experiment on focal variable (3 points)
Interpreting data	- Based on the gathered data, a correct **inference** was made - The **explanation of the inference** refers to the data or outcome variable - The data on which the inference was based are **described** or - the outcome is **explained**	Part 1: Do you see a difference in the table? **yes/no** 1 point if answer aligns with data; 0 if not Part 2: How do you know? ‘the number of bounces is different’ (1 point: refers to outcome variable) ‘on a hard surface the ball makes 5 more bounces than on a soft surface’ (2 points: describes data and refers to variable)
Drawing conclusions	- The **effect that was found** was described - The **direction** of the effect was described - All **variables involved** were described	‘It makes a difference’ (1 point; only if this was really found) ‘The surface matters for the number of bounces’ (2 points) ‘The ball made more bounces on a hard surface’ (3 points)

A set of 86 randomly selected worksheets was coded by a second independent rater; the intraclass correlation (ICC) was calculated as a measure of interrater reliability. The ICC was high for all component skills: hypothesizing (0.91, *p* < 0.001), experimenting (0.82, *p* < 0.001), interpreting data (0.94, *p* < 0.001), and drawing conclusions (0.89, *p* < 0.001). Differences in interrater agreement were resolved through discussion. If children were present during all lessons, nine worksheets would be available. In practice, some children missed one lesson and some worksheets got lost in the classroom. As a result, between six and nine worksheets were available per child.

### Procedure

The study was carried out over a period of 6 weeks according to the setup outlined in Figure 1. During the 1st week, all children made the pre-test in a whole-class test setting. In weeks 2–6, children participated in five 1-hr lessons taught by the first author. Due to time constraints, the final lesson included the post-test and, hence, contained only one small-group inquiry. As the study did not aim to compare different instructional treatments, all children received the exact same lessons.

## Results

**FIGURE 2:**
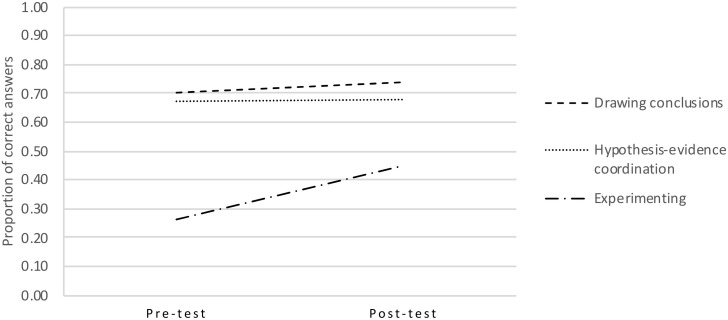
Pre- and post-test scores per component scientific reasoning skill.

Standardized progress monitoring data of reading comprehension and mathematics were obtained from 138 children (see Table 2); their pre- and post-test scores on the SRI are shown in Figure 2 and Table 3. These data show that, overall, children improved in scientific reasoning, but improvement rates differed among component skills. In order to explore these differences in scores and establish their relations with reading comprehension and mathematical skillfulness, a repeated measures multivariate analysis of covariance (MANCOVA) was carried out with time and component skill as within-subject variables, and reading comprehension level and mathematics level as between-subject covariates.

**TABLE 2:** Descriptive statistics on reading comprehension and mathematics.

	Level				
	I	II	III	IV	V
Reading comprehension	30.4%	25.4%	19.6%	15.2%	9.4%
Mathematics	26.1%	17.4%	25.4%	18.8%	12.3%

**TABLE 3:** Pre- and post-test scores on the Scientific Reasoning Inventory.

	Pre-test		Post-test		Gain	
	*M*	*SD*	*M*	*SD*	*M*	*SD*
Hypothesis-evidence coordination	0.67	0.23	0.68	0.27	0.01	0.21
Experimenting	0.26	0.22	0.45	0.35	0.20	0.38
Drawing conclusions	0.70	0.22	0.74	0.24	0.04	0.25
Overall	0.56	0.15	0.63	0.22	0.07	0.17

*Scores are reported as proportion of correct answers.*

### Development and Prediction

Multivariate test results showed a main effect of time, Wilk’s λ = 0.801, *F*(1, 134) = 33.393, *p* < 0.001 and skill, Wilk’s λ = 0.883, *F*(1, 134) = 8.840, *p* < 0.001. In addition to these main effects, an interaction was found between time and skill, Wilk’s λ = 0.796, *F*(2, 133) = 17.014, *p* < 0.001, indicating asynchronous development of the component skills over time. Lastly, three-way interactions were found between time, skill and reading comprehension, Wilk’s λ = 0.943, *F*(2, 133) = 4.040, *p* = 0.020, and time, skill and mathematical skillfulness, Wilk’s λ = 0.907, *F*(2, 133) = 6.842, *p* = 0.001, indicating that both reading comprehension and mathematical skillfulness explain variation in development of the component skills throughout the lesson series.

**FIGURE 3:**
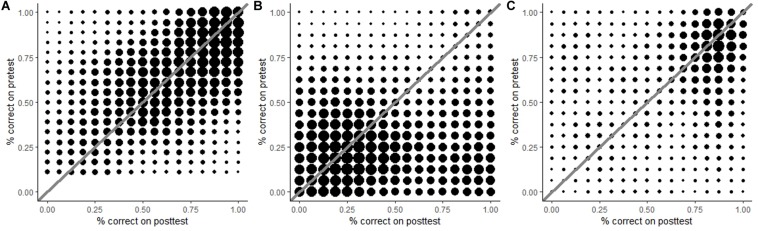
Density plots for hypothesis-evidence coordination **(A)**, experimenting **(B)**, and drawing conclusions **(C)**. For all component skills, most scores cluster around the diagonal, indicating limited growth. For experimenting, a second cluster can be seen in the lower right corner, indicating a large improvement for a small group of children.

Both the data in Table 3 and the significant time × skill interaction suggest that there may be subgroups of children who learned more than others. To examine this possibility, children’s change in scores from pre- to post-test were visualized in density plots for each component skill (Figure 3). In these plots, the diagonal line stands for ‘no development’; the area above the diagonal represents a decline in score, and the area below the diagonal indicates progress. For hypothesis-evidence coordination and drawing conclusions, most dots accumulate around the diagonal, meaning that children generally made little progress in these skills. A similar pattern was found for experimenting, except that there was an additional group of dots in the lower right corner. Thus, although the majority of children hardly progressed in experimenting, a small group did. It is noteworthy that the two areas are horizontally aligned. This means that some children who scored very low on the pre-test still learned to experiment very well.

In order to further explore the three-way interactions, parameter estimates were requested for pre- and post-test scores as well as for the gain scores (Table 4). These showed that for hypothesis-evidence coordination, both reading comprehension and mathematical skillfulness related to pre- and post-test scores. The predictors did not relate to gain scores on this skill, likely because there was very little progress. For experimenting, pre-test scores were not related to reading comprehension or mathematics, while post-test and gain scores were. Drawing conclusions was not related to children’s reading comprehension or mathematical skillfulness at all.

**TABLE 4:** Parameter estimates for interaction effects.

	Pre-test		Post-test		Gain scores	
	β	*p*	β	*p*	β	*p*
**Hypothesis-evidence coordination**						
Reading	0.133	<0.001	0.189	<0.001	0.056	0.179
Math	0.088	0.001	0.089	0.003	0.001	0.976
**Experimenting**						
Reading	0.024	0.591	0.277	<0.001	0.253	<0.001
Math	0.039	0.231	0.242	<0.001	0.202	<0.001
**Drawing conclusions**						
Reading	0.021	0.603	0.062	0.165	0.041	0.423
Math	0.030	0.324	0.039	0.235	0.009	0.808

*Previous analyses showed that gains in hypothesis-evidence coordination and drawing conclusions were not significant.*

### Key Learning Moments

The third research question addressed children’s learning process by identifying possible key learning moments during the lesson series. The worksheets children filled out during the lessons provided insight in this. A partial correlation between overall post-test scores (controlled for pre-test scores) and average worksheet scores was found, Spearman’s ρ = 0.408, *p* < 0.001, warranting further inspection of the process data summarized in Table 5. The partial correlation coefficients in this table show that the association between post-test and worksheet was consistent for some, but not all component skills. Specifically, hypothesizing and drawing conclusions (worksheets) were not related with any of the component skills measured by the Scientific Reasoning Inventory (SRI). Experimenting (worksheets) on the other hand did correlate with experimenting (SRI) as well as with hypothesis-evidence coordination (SRI). Interpreting data (worksheets) was associated with drawing conclusions (SRI).

**TABLE 5:** Average worksheet scores and partial Spearman’s rank correlations with post-test scores.

			Scientific Reasoning Inventory^1^		
			H-E coordination	Experimenting	Drawing conclusions
Worksheets	*M*	*SD*	ρ	ρ	ρ
Hypothesizing	1.40	0.57	0.122	0.145	0.136
Experimenting	1.96	0.63	0.308**	0.492**	0.120
Interpreting data	1.59	0.55	0.121	0.102	0.189*
Drawing conclusions	1.21	0.64	0.076	0.088	0.034

Worksheet scores ranged from 0 to 3 points. ^1^Post-test scores, controlled for pre-test; *p ≤ 0.05; **p ≤ 0.001.

**FIGURE 4:**
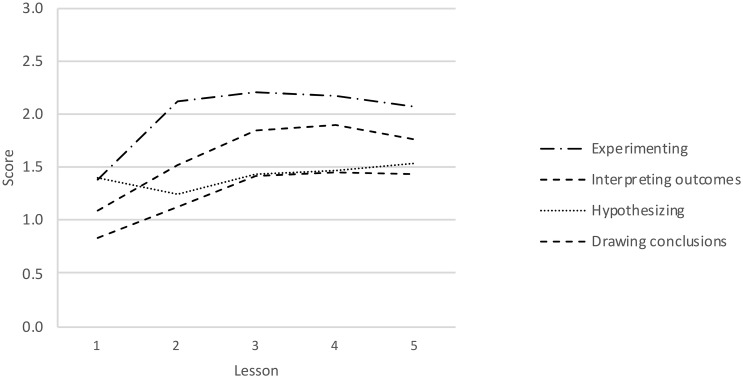
Worksheet scores per component scientific reasoning skill in each lesson.

In addition to correlations between children’s in-class performance and their achievements on the SRI, children’s progress throughout the lessons was examined. First, visual inspection of the line graphs in Figure 4 helped determine whether progress was actually made, and if so, at which moment(s) during the lesson series this growth was most pronounced. For hypothesizing, the slope appears more or less level, indicating no or very moderate improvement. Progress in the other three component skills appears to be made between the first and third lesson, after which it levels off. The first, third and fifth lesson were therefore used as anchor points in children’s developmental trajectories. Repeated measures ANOVAs were performed to compare the scores on each component skill at these three timepoints. As expected based on the line graph, no main effect was found for hypothesizing, Wilk’s λ = 0.984, *F*(2, 115) = 0.932, *p* = 0.400, while significant within-subject differences were found for experimenting, Wilk’s λ = 0.565, *F*(2, 115) = 44.287, *p* < 0.001, interpreting data, Wilk’s λ = 0.659, *F*(2, 115) = 29.706, *p* < 0.001, and drawing conclusions, Wilk’s λ = 0.770, *F*(2, 115) = 17.145, *p* < 0.001. Pairwise comparisons across the three timepoints were made to pinpoint when learning took place. The results in Table 6 show that for experimenting, interpreting data and drawing conclusions, significant progress was made between lessons 1 and 3, but not between lessons 3 and 5.

**TABLE 6:** Key learning moments in children’ scientific reasoning skills inferred from their worksheet scores.

	Lesson	*M*	*SD*	Change^1^	p_*change*_
Hypothesizing	1	1.35	0.90		
	3	1.45	0.77	0.10	0.574
	5	1.49	1.10	0.04	0.978
Experimenting	1	1.37	0.80		
	3	2.19	0.88	0.82	< 0.001
	5	2.07	1.03	–0.12	0.387
Interpreting data	1	1.09	0.97		
	3	1.85	0.74	0.76	< 0.001
	5	1.78	0.83	–0.07	0.734
Drawing conclusions	1	0.84	1.00		
	3	1.44	0.84	0.60	< 0.001
	5	1.45	1.13	0.01	1.000

*Worksheet scores ranged from 0 to 4 points; ^1^compared to previous.*

To assess whether improvement of the worksheet scores could be explained by children’s reading comprehension and mathematical skillfulness, a 3 (lessons) × 4 (skills) MANCOVA was performed, with reading comprehension and mathematical skillfulness as covariates. Multivariate test results showed no significant three-way interaction between lesson, skill and reading comprehension, Wilk’s λ = 0.908, *F*(6, 109) = 1.833, *p* = 0.099. Between lesson, skill and mathematical skillfulness a three-way interaction was found, Wilk’s λ = 0.881, *F*(6, 109) = 2.461, *p* = 0.029. However, further analysis of each component skill did not yield significant interactions between time and mathematical skillfulness. Thus, although mathematical skillfulness appears to predict progress in some component skills of scientific reasoning, this effect is not large enough to detect with more specific analyses.

## Discussion

The main purpose of this study was to investigate how children’s scientific reasoning develops during an inquiry-based science unit, and which cognitive characteristics predict progress of its component skills. Process data gathered during the lessons was analyzed to identify key moments during the lesson series when this progress was most pronounced. The findings, in short, point to a differential instructional effectiveness which should be taken into account in designing future adaptive learning arrangements.

Considerable diversity was observed in children’s proficiency in and learning of scientific reasoning. Although there were significant overall gains on the SRI, this improvement did not apply equally to all component skills. Specifically, children advanced their experimenting skills, but not their ability to coordinate hypotheses with evidence and draw conclusions. Overall gains were explained by children’s reading comprehension and mathematical skillfulness, as was their progress on experimenting skills and post-test performance on the hypothesis-evidence coordination items. However, both predictors explained neither progress nor proficiency in drawing conclusions. Finally, children’s worksheets evidenced progress over the lessons on experimenting, interpreting data and drawing conclusions, but not on hypothesizing. Most progress was made during the first half of the lesson series. These main outcomes of the study are discussed further below.

### Predicting Progress in Scientific Reasoning

The first two research questions focused on children’s progress on the component skills of scientific reasoning, with reading comprehension and mathematical skillfulness as predictors. Very little to no progress was expected to occur for hypothesis-evidence coordination and drawing conclusions, which indeed turned out to be the case. Although these component skills are often deemed more difficult than experimenting, pre-test scores were rather high in the current study. Still, the complete absence of progress is somewhat remarkable and suggests that both skills are not only hard to perform but also difficult to improve. No interactions were found between the predictor variables and progress on either hypothesis-evidence coordination or drawing conclusions, but children’s *proficiency* in hypothesis-evidence coordination interacted with reading comprehension and mathematical skillfulness on both pre- and post-test. This result seems understandable because the scale combined items that tap into the ability to identify appropriate research questions and interpret data, which are component skills that were expected to interact with both predictor variables.

Our hypotheses regarding experimenting were twofold: we either expected to find high pre-test scores and little progress, or low pre-test scores and substantial growth. Evidence was found for the latter hypothesis, although post-test scores for experimenting were lower than those for hypothesis-evidence coordination and drawing conclusions. This is noteworthy because experimenting is often regarded as one of the least difficult scientific reasoning skills to learn. The large standard deviations on the post-test imply that some children had improved more than others, which was confirmed by the interactions of both the post-test scores and progress with reading comprehension and mathematical skillfulness. In combination with the density plots shown in Figure 3, it therefore seems plausible that some, but not all children developed adequate experimentation strategies through structured, repeated practice. Informal observations during the lessons further indicated that some children realized that the research question could not be answered based on a confounded experiment. As the worksheets did not explicitly link experimental design to drawing conclusions, conceptualizing this connection required unsupported inferencing. The significant impact of children’s language and math skills suggests that only children with relatively high intellectual abilities were able to make this inference.

### Progress on Scientific Reasoning During the Lessons

Children’s entries on the worksheets were analyzed to unveil key moments in the learning process where marked progress in scientific reasoning was made. Notable improvements in experimenting, interpreting data and drawing conclusions occurred between lesson 1 and lesson 3, whereas no progress in hypothesizing was made over the five lessons. The latter result may be due to the fact that, unlike the other component skills, children’s hypotheses were rarely addressed during the whole-class discussions. Another possibility is that hypothesizing is easier if one has a theoretical basis on the topic of inquiry (Koslowski et al., 2008), which the children in our study had not or to an insufficient degree. The lack of growth in hypothesizing skills might be attributable to a combination of these factors.

Progress on the other component skills occurred between lesson 1 and lesson 3. Interestingly, children’s performance stabilized after the third lesson, despite the absence of a ceiling effect. This raises the question as to why progress leveled off before mastery was reached. A possible answer lies in the design principles underlying the lesson series. Both the lessons and the worksheets were highly structured (guided inquiry, Bell et al., 2005) but contained few explicit directions and explanations. The available implicit guidance enabled children to improve their scientific reasoning to some extent, meaning that additional growth may require additional guidance, extended practice, or both.

Using a combination of instructional support measures might help sustain children’s progress beyond the third lesson. Previous research comparing open and guided inquiry to direct instruction (e.g., Alfieri et al., 2011; Wagensveld et al., 2014; Vorholzer et al., 2018; Wagensveld et al., 2014) indicated that open inquiry was often ineffective, whereas guided inquiry or direct instruction yielded higher learning outcomes. Using data from the 2015 Trends in International Mathematics and Science Study (TIMSS), Teig et al. (2018) also concluded that inquiry can be an effective approach, but only when combined with other, more explicit forms of guidance. Along these lines, more specific directions by the teacher or through the worksheets could have caused the children in our study to make significant progress on hypothesizing and to fully master the skills of experimenting, interpreting data and drawing conclusions. What these instructions should entail and how they are best combined with the scaffolding offered by the worksheets are interesting questions for future research.

### Toward Adaptive Science Instruction

The present findings suggest that some children need little support to improve their scientific reasoning skills, whereas others seem to require more or more specific guidance. The worksheet data show that children improved in all scientific reasoning skills except hypothesizing; this progress was often modest and occurred in the first half of the lesson series. In order to help children further improve their scientific reasoning, we have three suggestions. First, guidance could be increased on component skills that are particularly difficult to learn, such as hypothesizing. Second, considering the relations found between scientific reasoning and children’s reading comprehension and mathematical skillfulness, progress monitoring data of these school subjects can help teachers to adapt their science lessons in advance, for instance by planning to offer more or more explicit guidance to children with lower levels of reading comprehension. Third, monitoring in-class performance can inform teachers when children need additional support. Using this information to make instant adjustments above and beyond the pre-planned adaptations could be a crucial next step in the improvement of elementary science education.

### Strengths and Limitations

On the positive side, this study examined multiple component skills of scientific reasoning under rather uniform conditions. As argued by Koerber and Osterhaus (2019), cross-study comparisons of proficiency and developmental growth in distinct scientific reasoning skills are likely confounded by differences in learner characteristics and task settings. Their plea for more comprehensive investigations of scientific reasoning was met here, and allows for more valid conclusions on the relative ease or difficulty with which individual scientific reasoning skills are acquired during elementary science lessons.

Another asset of this study is the use of two complementary data sources: the SRI and the worksheets. The origin of an instrument (existing or made for the study) can affect the outcomes (Schwichow et al., 2016). So in order to shed more light on children’s science learning in regular classrooms, but without compromising experimental validity, we combined scores on the experimentally valid SRI, administered in a test setting, with more ecologically valid data from the worksheets children filled out during the lessons.

Although this approach yielded valuable insights in the development of some scientific reasoning skills, an unforeseen discrepancy between these two data sources arose. Although the SRI and the worksheets both targeted the same component skills (hypothesizing, experimenting, interpreting data and drawing conclusions), factor analysis of the SRI-items in both the validation study and the current study required us to combine two of these skills in a single scale. This complicated the comparison of children’s scores on the worksheets and the SRI.

This measurement inconsistency is inconvenient because different proficiency patterns emerged for the two test modalities, which are now difficult to explain. While the worksheets outcomes followed the hypothesized proficiency pattern, with highest scores for experimenting and lowest for hypothesizing, the SRI scores for hypothesis-evidence coordination and drawing conclusions were high and scores on experimenting were low. Strong claims about what accounted for these discrepancies cannot be made, but there are several possible explanations.

First, differences in test item format may have played a role. Previous studies showed that the type of data greatly influences the ease of interpretation (Kanari and Millar, 2004; Masnick and Morris, 2008). The hypothesis-evidence coordination items on the SRI featured unambiguous, dichotomous outcomes that were relatively easy to interpret, whereas the data children gathered during the lessons were continuous and more messy. Although both called upon children’s ability to interpret data, requirements on the SRI were relatively limited. The high scores on hypothesis-evidence coordination and drawing conclusions suggest that the SRI taps children’s basic proficiency in these component skills, whereas the worksheet provides a more authentic assessment. Secondly, surface characteristics may have limited comparability too (Stiller et al., 2016). For example, longer questions and data tables (as used on the SRI scale hypothesis-evidence coordination) can decrease difficulty, whereas the longer response options (which were used on the worksheets for hypothesizing and drawing conclusions) may increase difficulty.

Finally, reliability of the experimenting scale of the SRI pre-test was low. This was probably caused by the fact that children did not have much experience with experimenting, and because the item format was relatively difficult for them. As a result, the range of scores on the pre-test was small and this limited variability may have affected Cronbach’s α.

### Implications and Directions for Further Research

Although the present study provides initial directions for adaptive science education, future research is needed to assess the effectiveness of these adaptions. This and other studies show that scientific reasoning can be taught to children of all cognitive levels (Zohar and Dori, 2003), yet less is known about how the needs of individual children in a class are best met. So although our findings indicate that teachers can base instructional adaptations on children’s proficiency in reading and math, research should investigate additional ways to adapt instruction in scientific reasoning.

Although the relationship between reading comprehension and scientific reasoning is well-established and caused some to conclude that scientific reasoning is linguistic in nature (Van de Sande et al., 2019), the relation between mathematical skillfulness and scientific reasoning has only recently been shown (Koerber and Osterhaus, 2019). The current study confirms that such a relationship exists. Acknowledging the impact of mathematical skillfulness is important for the effective teaching of scientific reasoning, which can be more thoughtfully designed bearing this information in mind.

## Conclusion

Fifth-graders generally improved in scientific reasoning during a 5-week inquiry-based lesson series. They made progress in all constituent skills except hypothesizing, mainly during the first half of the lesson series, and consolidated their increased experimentation skills on the post-test. Reading comprehension and mathematical skillfulness accounted for part of the variance in children’s progress and proficiency scores, and offer fertile grounds for adaptivity. However, more research is needed to fully grasp the individual variation in children’s science learning and explore ways to accommodate these differences. The outcomes of these studies contribute to the design of effective elementary science education for all.

## Data Availability Statement

The datasets generated for this study are available on request to the corresponding author.

## Ethics Statement

The studies involving human participants were reviewed and approved by Ethics Committee of the Faculty of Social Sciences at Radboud University (ECSS). Written informed consent from the participants’ legal guardian/next of kin was not required to participate in this study in accordance with the national legislation and the institutional requirements.

## Author Contributions

ES, IM, and AL contributed to the conception and design of the study. ES collected and analyzed the data. ES wrote the first draft of the manuscript and prepared the tables and figures. IM and AL gave feedback on all parts of the manuscript draft and rewrote several sections. All authors contributed to manuscript revision, read and approved the submitted version.

## Conflict of Interest

The authors declare that the research was conducted in the absence of any commercial or financial relationships that could be construed as a potential conflict of interest.
