# Supplementary material for: Individual Differences in Children’s Development of Scientific Reasoning Through Inquiry-Based Instruction: Who Needs Additional Guidance?
Source: Front Psychol. 2020 May 14;11:904. doi: 10.3389/fpsyg.2020.00904 (PMC7241249; doi:10.3389/fpsyg.2020.00904)
Supplement: Supplementary file 1 [file Data_Sheet_1.pdf]

Support levels during inquiry sessions

Hypothesizing

1. What are you going to change? (identify the variable of interest)
    a. You can change **A**, **B** or **C**
    b. Give answer
2. How can you change **A**? (determine the levels of the variable of interest)
    a. Which different **A**'s can you use in this inquiry?
    (e.g. if rope length is the variable of interest, you can choose long or short)
    b. Give answer
3. What are you going to measure? (identify output variable)
    a. Will you measure **X**, or **Y**, or something else?
    b. Give answer
4. Repeat original question: what do you think will happen with **X** if you change **A?**

Experimenting

1. What are you investigating? (identify the variable of interest)
    a. You can investigate **A**, **B** or **C**, which one are you investigating on this worksheet?
    b. Give answer
2. What should you do with **A** to find out whether **A** makes a difference for **outcome X**?
    a. You can change **A** or keep it the same, what do you think is best?
    b. Give answer
3. What should you do with **B** and **C** to investigate fairly whether **A** makes a difference for **outcome X**?
    a. You can change **B** and **C** or keep them the same, what should you do for a fair investigation?
    b. Give answer

Inferencing 1 (single comparison)

1. What did you measure?
    a. Did you measure **X**, or **Y**, or something else?
    b. Give answer
2. What was the outcome of **experiment 1**? and of **experiment 2**?
    a. Point out what the child wrote down
    b. Give answer
3. Where the outcomes the same or different?
    a. Is **outcome 1** the same as **outcome 2**?
    b. Give answer

Inferencing 2 (multiple comparison)

1. For every time you performed the experiment, draw a circle around the largest outcome
    a. The firs time you did the experiment, was the outcome of **experiment 1** the largest or the outcome of **experiment 2**? Draw a circle around the largest one.
        i. Ask child to do the same for each replication
        ii. Repeat question 1a for each of the replication
    b. Give answer
2. Did you draw more circles for **experiment 1** or for **experiment 2**?
    a. How many circles did you draw for **experiment 1**? And how many for **experiment 2**?
    b. Give answer
3. Was the difference very large, or not so large?

Conclusion

1. What did you investigate? (identify the variable of interest)
    a. Did you investigate **A**, **B** or **C** on this worksheet?
    b. Give answer
2. What was the **value** of **A** in experiment 1? And in **experiment 2**? (determine the levels of the variable of interest & how they were set in the experiments this child performed)
    a. Stimulate to look it up
    b. Point out/give answer
3. Were the outcomes for **experiment 1** different than those for **experiment 2**?
    a. Stimulate to look it up
    b. Point out/give answer
