# Supplementary material for: Individual Differences in Children’s Development of Scientific Reasoning Through Inquiry-Based Instruction: Who Needs Additional Guidance?
Source: Front Psychol. 2020 May 14;11:904. doi: 10.3389/fpsyg.2020.00904 (PMC7241249; doi:10.3389/fpsyg.2020.00904)
Supplement: Supplementary file 2 [file Data_Sheet_2.pdf]

number:

RESEARCH

Does the length of the rope affect the period of a swing?

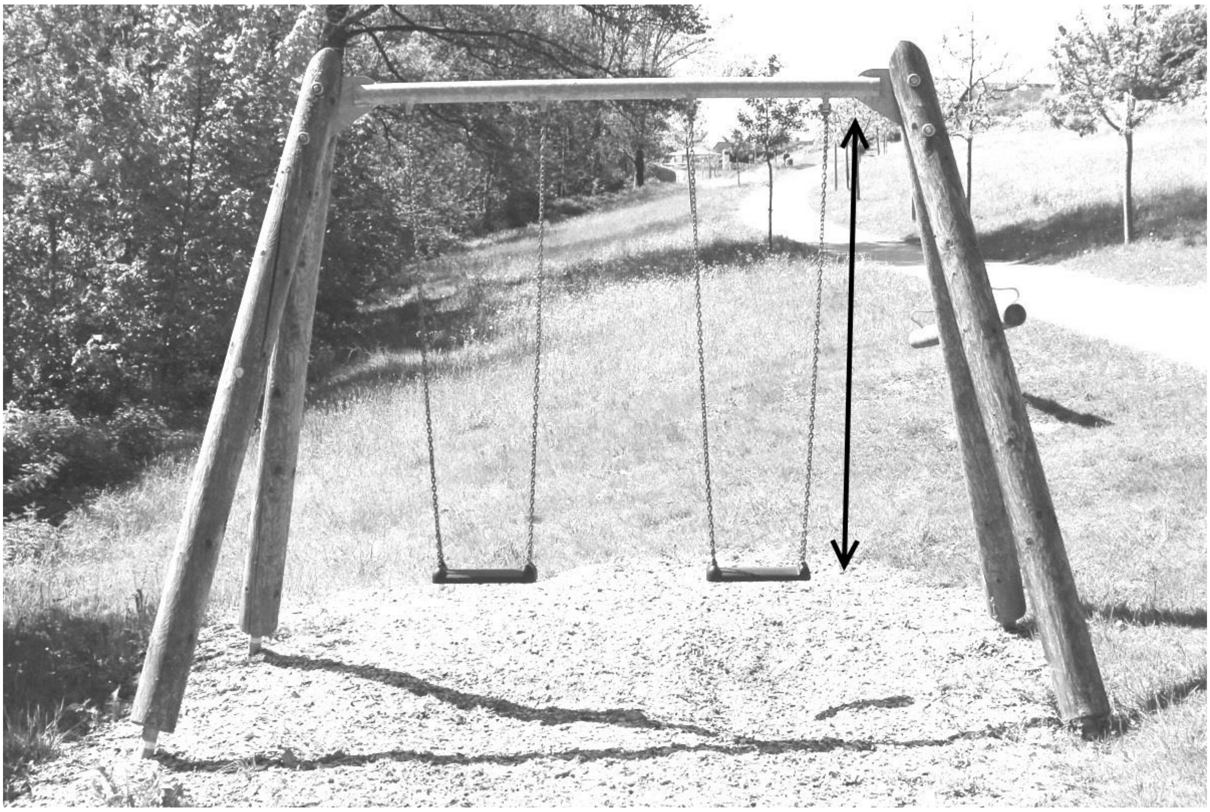

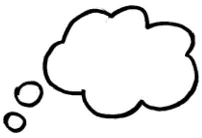

Expectations

What do you think will happen to the period of a swing if you change the length of the rope?

I think ……

………

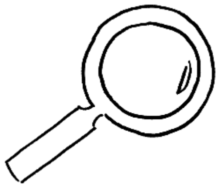

Experiment

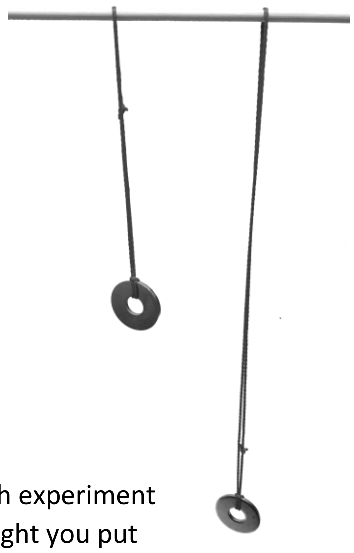

Think up two experiments to check if the length of the rope affects the period. For each experiment you have to choose the length of the rope, how far you pull it aside and how much weight you put on the pendulum

Experiment 1A		Experiment 1B	
Rope length:	....	Rope length:	....
How far pulled aside:	....	How far pulled aside:	....
Weigth:	....	Weigth:	....

Perform each of the experiments together with an assistant. Take care to measure the same thing each time: how long does it take for the pendulum to swing 5 times?

Experiment 1A		Experiment 1B	
Outcome:	....	Outcome:	....

Do you see a difference between a short and a long rope?

yes/no

To be more sure, you can repeat your experiments:

Experiment 1A

Outcome 2nd trial: ………………..

Outcome 3rd trial: ………………..

Experiment 1B

Outcome 2nd trial: ………………..

Outcome 3rd trial: ………………..

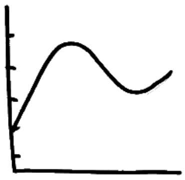

Outcomes

Copy all outcomes of all trials in the table below (do not forget your first trial!). That way, all outcomes are conveniently grouped together.

	**Experiment 1A**	**Experiment 1B**
1st trial	…………	…………
2nd trial	…………	…………
3rd trial	…………	…………

Do you see a difference between a short and a long rope in the table?

yes/no

How do you know?

……

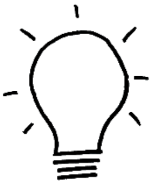

Conclusion

You can now answer the research question:

Does the length of the rope affect the period of a swing?

My research shows …….

………
