# Supplementary material for: Individual Differences in Children’s Development of Scientific Reasoning Through Inquiry-Based Instruction: Who Needs Additional Guidance?
Source: Front Psychol. 2020 May 14;11:904. doi: 10.3389/fpsyg.2020.00904 (PMC7241249; doi:10.3389/fpsyg.2020.00904)
Supplement: Supplementary file 3 [file Data_Sheet_3.pdf]

Appendix 3: example items for standardized tests

Reading comprehension

Original item:

Lees eerst Voorbeeldtekst 1 in het Tekstboekje: Noordpool en Zuidpool. Deze tekst bestaat uit drie alinea's. Maak daarna voorbeeldopgave V1.

Sem maakt een tabel bij de tweede en derde alinea. Deze tabel is nog niet helemaal af.

	**Noordpool**	**Zuidpool**
**Dieren**	ijsberen<br>zeehonden	pinguïns<br>zeehonden
**1**	Inuit	onderzoekers

V1 Wat moet hij op plaats 1 zetten?

A Antarctica
B IJsberen
C Mensen
D Pinguïns

Voorbeeldopgave groep 6, samenvatopgave

Translation:

Read example text 1 in the Text booklet: North pole and South pole. This text consists of three paragraphs. After reading, make example item V1.

Sem makes a table to summarize the second and third paragraph. The table is not fully finished.

	**North pole**	**South pole**
**Animals**	ice bears<br>seals	penguins<br>sea lions
**1**	Inuit	researchers

V1 What should he put on place 1?
A Antarctica
B Icebears
C People
D Penguins

Retrieved on 19/03/2020 from https://www.cito.nl/-/media/files/ve-en-po/voorbeeldopgaven-producten/cito-leerlingvolgsysteem-lvs-begrijpend-lezen-voorbeeldopgaven.pdf?la=nl-NL

Mathematical skillfulness

Original item 1: no context

199 + 199 + 199 = ________

Voorbeeldopgave groep 6, kale opgave

Original item 2: contextualized

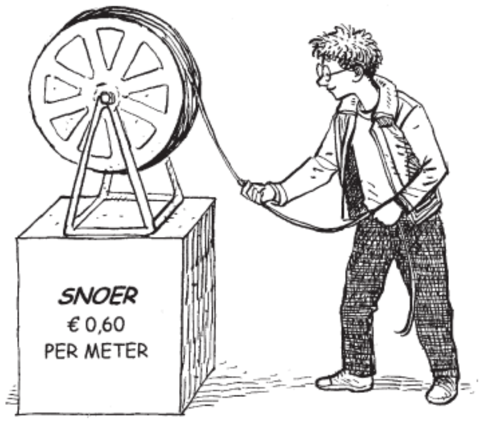

Jesper koopt 2 meter en 50 centimeter van dit snoer.

Hoeveel euro moet Jesper betalen?

€ ______

Voorbeeldopgave groep 6, contextopgave

Translation:

Jesper buys 2 meters and 50 centimeters of this cable.

How much does Jesper need to pay?

€ ________

Retrieved on 19/03/2020 from https://www.cito.nl/-/media/files/ve-en-po/voorbeeldopgaven-producten/cito-leerlingvolgsysteem-lvs-rekenen-wiskunde-voorbeeldopgaven.pdf?la=nl-NL
